# Craniofacial anomalies associated with hypospadias. Description of a hospital based population in South America

**DOI:** 10.1590/S1677-5538.IBJU.2015.0568

**Published:** 2016

**Authors:** Nicolas Fernandez, Rebeca Escobar, Ignacio Zarante

**Affiliations:** 1 Hospital Universitario San Ignacio Pontificia Universidad Javeriana - Urología - Genética Bogota, Colombia; 2 Universidad del Rosario - Epidemiología, Bogotá, Colombia; 3 Hospital Universitario San Ignacio Pontificia Universidad Javeriana - Genética, Bogotá, Colombia

**Keywords:** Hypospadias, Craniofacial Abnormalities, South America, Population Groups

## Abstract

**Introduction:**

Hypospadias is a congenital abnormality of the penis, in which there is incomplete development of the distal urethra. There are numerous reports showing an increase of prevalence of hypospadias. Association of craniofacial malformations in patients diagnosed with hypospadias is rare. The aim of this study is to describe the association between hypospadias and craniofacial congenital anomalies.

**Materials and Methods:**

A retrospective review of the Latin-American collaborative study of congenital malformations (ECLAMC) data was performed between January 1982 and December 2011. We included children diagnosed with associated hypospadias and among them we selected those that were associated with any craniofacial congenital anomaly.

**Results:**

Global prevalence was 11.3 per 10.000 newborns. In this population a total of 809 patients with 1117 associated anomalies were identified. On average there were 1.7 anomalies per patient. Facial anomalies were present in 13.2%. The most commonly major facial anomaly associated to hypospadias was cleft lip/palate with 52 cases. We identified that 18% have an association with other anomalies, and found an association between craniofacial anomalies and hypospadias in 0.59 cases/10.000 newborns.

**Discussion:**

Hypospadias is the most common congenital anomaly affecting the genitals. Its association with other anomalies is rare. It has been reported that other malformations occur in 29.3% of the cases with hypospadias. The more proximal the meatus, the higher the risk for having another associated anomaly.

**Conclusion:**

Associated hypospadias are rare, and it is important to identify the concurrent occurrence of craniofacial anomalies to better treat patients that might need a multidisciplinary approach.

## INTRODUCTION

Hypospadias is one of the most common birth defect among newborn males worldwide. The prevalence rate varies greatly; in Australia it was calculated as 35.1 per 10.000 (1). In Colombia it affects around 1.7/1000 male live births (2). It is a congenital abnormality of the penis in which there is an incomplete development of the anterior urethra, resulting in the abnormal urethral opening on the ventral surface of the penis (3). Severity depends on the location of the urethral opening, which increases as the opening approaches the base of the penis (4). In most cases the cause is unknown; however, endocrine, environmental and genetic factors have been found to be involved in the etiology of hypospadias (3).

To date, there have been numerous reports indicating a higher incidence of associated anomalies in patients diagnosed with hypospadias. These abnormalities can be divided into genital, urinary tract and extra urinary; the latter including craniofacial and cardiothoracic alterations (5). Although most of the current data describe associations with urinary tract abnormalities, a study by Friedman et al. in 2008 showed an increased incidence of facial dysmorphic such as cleft palate, microphthalmos and high arched palate that had not been seen before.

Due to the fact that most of the associations of hypospadias reported in the literature have been presented as clinical case reports, it is difficult to establish the clinical repercussions of these associations and their epidemiology. This has created the need for population-based epidemiologic data in order to have a better understanding of the malformation phenotypes that co-occur with hypospadias (3). Furthermore, studies regarding co-occurring malformations can aid in the discovery of risk factors and provide new insight into the etiology of these malformations. The purpose of the present paper is to present the description of a large-scale multicenter study presenting information about the co-occurrence of hypospadias and craniofacial anomalies.

## MATERIALS AND METHODS

### Database description

Our study is based on the Latin-American collaborative study of congenital malformations (ECLAMC) initiative which is a multicenter international collaboration designed to identify associated risk factors and potential causes of congenital anomalies (CA). As mentioned on the ECLAMC methodology we followed a nested case-control design, analyzing information forwarded from each participating center. A retrospective review of data was performed between January 1982 and December 2012. The aim of our search included children diagnosed with associated hypospadias.

### Data collection and Quality Management

Data collection followed a standardized methodology for the entire study period. A daily surveillance was conducted on each participating center looking for detectable CA in all newborns. For every detected case, the following information was collected: mother's demographic data, prenatal and delivery information, and exposure to medications and toxics during pregnancy. This process was done by trained personnel in the ECLAMC methodology. For every enrolled case the immediate next newborn of the same sex was included as a control, collecting the same information.

### Inclusion Criteria

Hypospadias (IH) cases were strictly defined as newborns with an ectopic urethral meatus located along the ventral aspect of the penis. Depending on the location, these were further categorized as glanular (glans), coronal, penile and scrotal (6). Megameatus intact prepuce variants were included in the glanular hypospadias. Associated scrotal findings were also recorded. Associated anomalies were separately labeled. Each one of the associated anomalies was described in detail following the ECLAMC protocol for each of the different anomalies. Specifically for the craniofacial anomalies, an anatomical classification was followed according to the ECLAMC's methodology.

#### Statistical analysis

For all cases we analyzed the distribution of each abnormality and its association to the grade of hypospadias. Also for the demographic information, we compared non-associated cases of hypospadias to co-occurring hypospadias with craniofacial anomalies. In order to standardize the assessment, we segregated associated anomalies by affected systems: genitourinary tract (GUT), gastrointestinal tract (GIT), limbs, facial anomalies (FA), cardiovascular (CV) and nervous system (CNS), abdominal wall (ABD), and others.

## RESULTS

During the analysis period, a total of 159 centers in 6 countries participated in the study. The surveillance was conducted on 4.020.384 newborns; from these a total of 4.537 hypospadias cases were detected, with a global prevalence of 11.3 per 10.000 newborns. A total of 809 patients with 1117 associated anomalies were identified. On average there were 1.38-1.7 anomalies per patient. Facial anomalies were present in 13.2%.

When analyzing and comparing the severity of hypospadias and its relation to other anomalies, we found that proximal ones were significantly associated to other anomalies (penile hypospadias RR=1.64 [95%CI=1.33–2.03] and scrotal hypospadias RR=2.49 [95% CI 1.80– 3.47]). The distal cases failed to show association. No differences were identified when comparing non-associated to co-occurring craniofacial – hypospadias cases with regards to mother's age, gestational age, weight at birth.

In this population, 5128 cases of cleft lip/palate were identified, with a prevalence 12.2 cases per 10.000 newborns associated to hypospadias. For micrognatia a rate of 74 cases per 10.000 newborns was identified. 11% were associated to other anomalies, and the cases of hypospadias were selected.

The facial anomalies that were detected and its distribution according to the severity of hypospadias are shown in Table-1. The most commonly major facial anomaly associated to hypospadias was cleft lip/palate with 52 cases.

**Table 1:** Associated anomalies and its distribution by severity of hypospadias.

Malformation	Glanular n(%)	Coronal n(%)	Penile n(%)	Scrotal n(%)	Total	Prev/1000	%
Cleft lip and/or palate	21	23	3	5	52	11.5	21.8
Micrognatia	15	21	7	3	46	10.1	19.2
Low ear implantation	19	18	5	1	43	9.5	18.0
Preauricular pit	16	16	3	0	35	7.7	14.6
Microtia	10	14	5	1	30	6.6	12.6
Microphthalmus	4	2	0	1	7	1.5	2.9
Preauricular fistula	1	6	0	0	7	1.5	2.9
Leucoma	4	0	0	0	4	0.9	1.7
Hyperthelorism	0	2	1	1	4	0.9	1.7
Blefaroptosis	1	1	1	0	3	0.7	1.3
Neonatal teeth	2	0	0	0	2	0.4	0.8
Single narine	0	1	0	1	2	0.4	0.8
Macroglosia	0	1	0	0	1	0.2	0.4
Microglosia	1	0	0	0	1	0.2	0.4
Prognatism	0	0	1	0	1	4.5	0.4
Retrognatia	0	1	0	0	1	0.2	0.4
**TOTAL (%)**	**94 (39.3)**	**106 (44.4)**	**26 (10.9)**	**13 (5.4)**	**239**	**52.7**	

## DISCUSSION

Although hypospadias is the most common congenital anomaly affecting the genitals, its association with other anomalies is rare. It has been previously reported that other malformations can coexist in 29.3% of the cases with hypospadias. In our group we identified that 18% have an association with other anomalies. There is limited data on malformation phenotypes that coexist with hypospadias (3–7). Associations such as cryptorchidism are common and belong to the gonadal dysgenesis syndrome. Also, associations with urinary tract anomalies as part of the WAGR syndrome are commonly seen. But it has been demonstrated that other affected organs or systems in association with hypospadias are rare (3).

After analyzing the severity of hypospadias and the different craniofacial anomalies we did not find any significant trends with regards to severity of the craniofacial anomaly and hypospadias severity. The distribution was very homogeneous being most frequently associated with distal hypospadias.

Information about craniofacial anomalies that co-occur with hypospadias is limited. In our population we found an association between craniofacial anomalies and hypospadias in 0.52 cases/1000 newborns. Nassar et al. (4) reported 0.3 cases per 10.000 newborns.

Not many syndromes have been described where the two systems are affected. It can be associated with MID1 gene defects which interacts with MID2 (8), and are implicated in midline anomalies (9, 10). The Toriello Carey syndrome has the co-occurrence of hypospadias and facial anomalies. Others are the Mowat-Wilson Syndrome, the Opitz-Kawegia Syndrome and the hypertelorism-hypospadias syndrome described in 1969 (11–17). In our population we identified 4 cases (1.7%) of patients diagnosed with hypertelorism whom also had hypospadias. Other authors have identified a prevalence of 15.3% of associated cases of hypospadias-hypertelorism (5). Of those, two were proximal. It seems that these cases are inherited as an X-linked or autosomal dominant trait (18).

Some authors have reported the association between deletion of 13.q33.2 and the presence of hypospadias and craniofacial anomalies. Unfortunately, our database does not include information about karyotype.

With regards to cleft lip, previous reports have shown an association prevalence with hypospadias of 5.6/1000 (19). In our population we identified a prevalence of 11.5/1000 cases of hypospadias. Although this is one of the malformations that is easily diagnosed, we do not consider that it can be overestimated in our population.

According to our experience, and many other authors, most of the congenital anomalies if detected and treated appropriately and soon enough, the future disabilities and prognosis will improve significantly. Most of the craniofacial anomalies and hypospadias need to be approached by experienced personal. For that reason, it is important for this patients to be evaluated by a multidisciplinary approach (20–23).

## CONCLUSION

Our novel results indicate the importance of evaluating thoroughly all patients diagnosed with hypospadias. The prevalence of craniofacial anomalies is not increased in patients with proximal hypospadias. Or the prevalence of other anomalies is not increased in hypospadias patients who have craniofacial anomalies. Although syndromic hypospadias are rare and represent less than 20% of cases, it is important to identify the co-occurrence of craniofacial anomalies because it might help the identification of syndromic hypospadias in order to give a better care for patients and their families.
